# A new scheme for knee joint CT image segmentation: A max-flow and watershed method driving semi-automatic CT knee bone segmentation

**DOI:** 10.1038/s41598-026-53446-y

**Published:** 2026-05-30

**Authors:** Huayu Fan, Zhenyan Guan, Chaofan Yang, Pei Liu, Xin Yang, Liang Kong, Xi Chen, Yanqing Wang, Rui Chen, Zebing Ma, Yuping Duan, Zhi-Feng Pang, Xiangyang Cao

**Affiliations:** 1 Luoyang Orthopedic Hospital of Henan Province (Orthopedic Hospital of Henan Province), Zhengzhou, China; 2 Henan University of Chinese Medicine, Zhengzhou, China; 3 Henan University, Zhengzhou, China; 4 Beijing University of Chinese Medicine, Beijing, China; 5 Zhengzhou University, Zhengzhou, China; 6 Fujian University of Traditional Chinese Medicine, Fuzhou, China; 7 Shanghai Jiao Tong University, Shanghai, China; 8 Hunan University of Chinese Medicine, Changsha, China; 9 Beijing Normal University, Beijing, China; 10 Henan Academy of Traditional Chinese Medicine Sciences, Zhengzhou, China

**Keywords:** Biological techniques, Computational biology and bioinformatics

## Abstract

Knee joint segmentation from CT images is critical for diagnosing and treating arthritis and other knee disorders. Manual segmentation is time-consuming and expert-dependent, highlighting the need for automated, accurate, and efficient methods. This study proposes a scheme for 3D CT knee-joint segmentation that integrates an adaptive weighted continuous max-flow algorithm with a graphical user interface (GUI). Input CT volumes are preprocessed to normalize intensities and improve robustness. Segmentation employs a continuous max-flow formulation with an adaptive weighting function to better capture weak or ill-defined edges in knee CT data. The watershed algorithm is used to resolve adhesions and separate contiguous structures. The approach supports semi-supervised interaction, allowing limited manual guidance when necessary. A graphical user interface (GUI) facilitates data input, interactive refinement, and result export. Performance was assessed on 18 real datasets using precision, sensitivity, and specificity, and was compared against four open-source segmentation tools. Across the 18 datasets, the proposed method achieved higher precision, sensitivity, and specificity than the evaluated open-source tools, demonstrating improved segmentation accuracy and robustness in knee CT images. The proposed semi-automated scheme yields high-precision, efficient knee joint segmentation from 3D CT, reducing manual effort and streamlining the clinical workflow. The improved accuracy and robustness have the potential to enhance diagnostic and treatment planning in orthopedic applications.

## Introduction

Knee joint disease is a prevalent clinical ailment that can have a significant impact on the health and well-being of patients. The CT image can aid in diagnosing knee diseases. Consequently, the question of bone segmentation has evolved into a significant concern within the domain of clinical applications^1,2^. To address this challenge, experts typically use a meticulous marking process, delineating the pixels and voxels within the target area. This approach is undertaken to ensure optimal segmentation accuracy. However, the manual labelling process is time-consuming, laborious, and requires professional expertise. Consequently, there is a necessity to develop a fully automatic, high-precision, and high-efficiency bone segmentation system to facilitate the clinical process. In this regard, numerous knee joint segmentation techniques have been proposed for clinical applications^3–6^, including the active contour model^7^, the atlas-based registration^8^, the neural network-based segmentation^9^, the fuzzy clustering^10^, the super-pixel method^11^ and the graph-cut method^12^, among others.

Although these automatic segmentation techniques have advanced medical imaging diagnostics, they often produce errors when image quality and contrast are poor. Kang et al.^13^ developed an automated framework for 3D segmentation of knee joints from volumetric CT images using a region-growing algorithm with local (adaptive) thresholding. However, this method required post-processing for the local boundary adjustment. Ababneh et al.^14^ proposed a fully automated, content-based system for segmenting the knee bone, utilizing magnetic resonance imaging as the primary data source. However, this system required users to provide numerous sparse labels. Eijnatten et al.^15^ proposed a global thresholding scheme for segmenting CT images. However, the implementation of this scheme necessitated extensive manual post-processing. Pacheco-Carrasco et al.^16^ proposed an intensity and histogram-based energy minimisation approach for the joint segmentation of bones and muscles. However, its computational efficiency is inferior to other algorithms. Besler et al.^17^ proposed a reliable femur segmentation method by utilizing prior sparse labels.

In the field of orthopaedics, the integration of machine learning algorithms for diagnosing knee joint diseases has precipitated a paradigm shift. As demonstrated in^18^, deep learning methods have the capacity to thoroughly analyse the characteristics of medical images and produce accurate results. This includes bone fragment segmentation^19^, segmentation of the proximal femur^20^, automated fractured bone segmentation and labelling^21^, and knee articular cartilage segmentation^22^. Nonetheless, deep learning methods have been criticized for their poor generalization to unseen data. Despite recent advances in deep learning (DL) having significantly advanced medical image segmentation through frameworks like TotalSegmentator^1^ and nnUNet^23^, their clinical adoption in specialised orthopaedic applications remains constrained by three fundamental challenges. Firstly, the data-intensive nature of DL models, which necessitate thousands of annotated cases, presents a significant challenge for rare pathologies such as severe knee adhesions, where the availability of sufficient training data is inherently limited. Secondly, the inherent ”black-box” nature of these systems engenders an interpretability gap, as clinicians require adjustable parameters for critical edge refinement in surgical planning. Thirdly, practical hardware limitations emerge when deploying computationally intensive 3D deep learning (DL) models on cost-sensitive clinical workstations that demand real-time inference capabilities. To address these limitations and enhance clinical translatability, our methodology is built on three synergistic design principles. First,the use of mathematically transparent algorithms enable user-adjustable segmentation. Second, data-efficient learning frameworks require minimal annotated cases. Third, the computationally optimised architecture that ensures real-time performance on standard clinical hardware.

The knee joints are lined with cartilage, and a joint capsule surrounds the entire joint. This results in weak boundaries between the cartilage and the rest of the joint. As previously discussed, despite significant advancements in knee joint segmentation, the challenge of delineating weak boundaries persists, impeding the attainment of precise segmentation outcomes. To achieve this objective, the present study proposes a novel methodology for segmenting the knee joint, based on 18 real data sets. The data sets under consideration contain the bones of one knee, double knees, femurs, and acetabular joints. The numerical implementations of our scheme have been compared with those of several other opening software programmes. The results indicate that our scheme is more robust and stable in terms of several quantitative indices. Moreover, our GUI enables optimal segmentation with minimal user input, requiring only the original data.

The remainder of this paper is structured as follows. The essential technologies of the knee joint segmentation scheme, including data preprocessing, segmentation, morphological processing, and the watershed method, are outlined in Section 2. As demonstrated in Section 3, the detailed workflow process is presented, and accurate results for segmenting some clinical knee CT images are provided. The GUI interface of the knee joint segmentation scheme, along with its operational dynamics, is delineated in Section 4. The conclusion of this paper is provided in Section 5.

## Methods

This section focuses on the arrangement of HU preprocessing to enhance the contrast of regions of interest (ROIs) in CT images. Following the initial processing stage, several suitable segmentation techniques are proposed for dividing CT images. Despite the plethora of methodologies available, including variation-based and deep learning methods, the focus remains on the max-flow method, the watershed method, and the supervised segmentation method. Subsequently, a workflow is presented for the proposed scheme.

### Data preprocessing

Contemporary 3D CT scans provide diagnostically crucial medical images. CT scanners have been demonstrated to be the optimal choice for segmenting imaging bony structures, a result of their capacity to provide superior hard tissue contrast and spatial resolution^24^. The raw CT data are typically stored in Digital Imaging and Communications in Medicine (DICOM) format^15^. Currently, routine diagnostic scan protocols typically feature slice thicknesses ranging from 0.6 mm to 2 mm and voxel sizes ranging from 0.2 mm to 0.6 mm. The Hounsfield unit (HU) is a dimensionless unit that is universally employed in CT scanning to express CT numbers in a standardised and convenient form. The brightness level of organs and tissues is reflected in CT images, which represent the degree of X-ray absorption. Black shadows indicate low-absorption areas, i.e., low-density areas, such as the lungs, while white shadows indicate high-absorption areas, i.e., high-density areas, such as bones. Density-related disparities manifest as pronounced variations in HU values across different bone types. This study was based on knee joint CT images, all of which were obtained after acquiring informed consent from the subjects. The presence of pathological changes in bone has been shown to have a detrimental effect on bone tissue density^23^. This, in turn, has been shown to have a subsequent effect on the HU value.

**Fig. 1:**
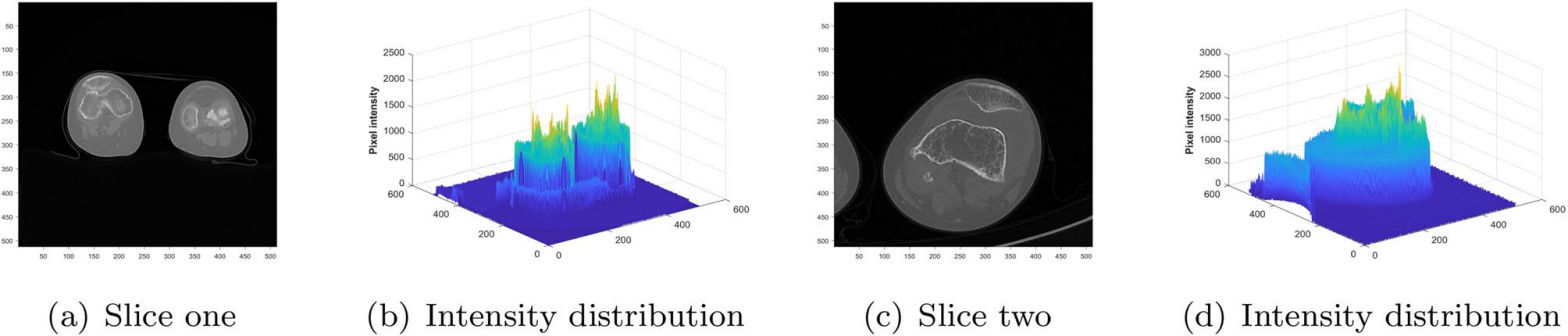
CT images of knee joint and the corresponding intensity distribution of pixel.

The issue of knee joint segmentation is addressed by leveraging the mineral composition of knee bones, which introduces contrast in X-ray images, as illustrated in Fig. 1. It is acknowledged that the incident beam intensity was not uniform, that the scanner calibration was inaccurate, and that there were differences in the patient’s age and gender. In order to address these issues, the HU pre-processing scheme was added to the range [300, 1600] for subsequent experiments.

The following subsection focuses on max-flow-based segmentation. During this period, significant attention has been devoted to investigating max-flow and min-cut models within a continuous framework for the image segmentation problem^25^. In^26^, Boykov et al. initially proposed the graph cut method for image segmentation, whereby the energy functional was combined with a region term and a boundary term. Nevertheless, the primary disadvantage inherent to the discrete configuration of the graph is frequently the result of the discrete grid, which gives rise to metrication errors. In this regard, Yuan et al.^27^ derived the dual of the energy function documented in^26^ and subsequently demonstrated that the dual is a max-flow problem in a continuous domain. However, the model utilised in^27^ exhibits a deficiency in the adaptation process. In this paper, a novel methodology is proposed to enhance the system’s robustness. This is achieved by introducing a weight function that can adaptively describe weak edges in knee CT images.

Let $\Omega \subset \mathbb {R}^{m\times n}$ be the continuous image domain of the knee CT image, the source flow $\mathcal {S}({\textbf {x}})$ directs from the source *s* to the image position ${\textbf {x}}$ and the sink flow $\mathcal {T}({\textbf {x}})$ directs from the source *t* to the image position ${\textbf {x}}$, $\textbf{q}({\textbf {x}})$ is the spatial flow at the image position ${\textbf {x}}\in \Omega$. Then we consider a new max-flow model as

1 $$\begin{aligned} \begin{aligned} \displaystyle \max _{\mathcal {S}({\textbf {x}}),\mathcal {T}({\textbf {x}}), {\textbf {q}}({\textbf {x}})}\hspace{10pt}&\displaystyle \int _{\Omega }\mathcal {S}({\textbf {x}})\textrm{d} {\textbf {x}},\\ \mathrm {s.t.}\hspace{30pt}&\mathcal {S}({\textbf {x}})\le C_{s}({\textbf {x}}),\hspace{3pt}\mathcal {T}({\textbf {x}})\le C_{t}({\textbf {x}}),\\&\Vert {\textbf {q}}({\textbf {x}})\Vert _{\infty }\le C({\textbf {x}})\boldsymbol{\alpha }({\textbf {x}}),\\&\textrm{div} {\textbf {q}}({\textbf {x}})-\mathcal {S}({\textbf {x}})+\mathcal {T}({\textbf {x}})=0, \end{aligned} \end{aligned}$$

where $C({\textbf {x}})$ is the capacity of the spatial flow ${\textbf {q}}({\textbf {x}})$, $C_{s}({\textbf {x}})$ and $C_{t}({\textbf {x}})$ denote the capacity of the source and sink flow, the edge indicator function $\boldsymbol{\alpha }({\textbf {x}})$ is defined by as

$$\begin{aligned} \boldsymbol{\alpha }({\textbf {x}})=\frac{1}{\sqrt{1+|\nabla I({\textbf {x}})|^{2}}}. \end{aligned}$$

Here $I({\textbf {x}}): \Omega \rightarrow \mathbb {R}$ denotes the image after the HU preprocessing. In addition, the values of $C_{s}({\textbf {x}})$, $C_{t}({\textbf {x}})$ and $C({\textbf {x}})$ depend on the value of the image $I({\textbf {x}})$, $\Vert \cdot \Vert _{\infty }$ denotes the $L^{\infty }$ norm.

For the optimization problem (1), we first introduce the multiplier function $w({\textbf {x}})$ for the equation constraint and then the corresponding augmented Lagrangian optimization problem can be rewritten as

2 $$\begin{aligned} \begin{aligned} \displaystyle \max _{\mathcal {S}({\textbf {x}}),\mathcal {T}({\textbf {x}}),{\textbf {q}}({\textbf {x}})} \min _{w({\textbf {x}})}\hspace{10pt}&\mathcal {L}(\mathcal {S}({\textbf {x}}),\mathcal {T}({\textbf {x}}), {\textbf {q}}({\textbf {x}});w({\textbf {x}})),\\ \mathrm {s.t.}\hspace{48pt}&\mathcal {S}({\textbf {x}})\le C_{s}({\textbf {x}})\hspace{3pt},\hspace{3pt}\hspace{3pt}\mathcal {T}({\textbf {x}})\le C_{t}({\textbf {x}}),\\&\Vert {\textbf {q}}({\textbf {x}})\Vert _{\infty }\le C({\textbf {x}})\boldsymbol{\alpha }({\textbf {x}}). \end{aligned} \end{aligned}$$

where the augmented Lagrangian function is defined by

$$\begin{aligned} \mathcal {L}(\mathcal {S}({\textbf {x}}),\mathcal {T}({\textbf {x}}),{\textbf {q}}({\textbf {x}});w({\textbf {x}})):=&\int _{\Omega }(1-w({\textbf {x}}))\mathcal {S}({\textbf {x}})+w({\textbf {x}})\mathcal {T}({\textbf {x}}) +w({\textbf {x}})\textrm{div} {\textbf {q}}({\textbf {x}})\textrm{d} {\textbf {x}} \\ &-\frac{\lambda _{1}}{2}\Vert \textrm{div} {\textbf {q}}({\textbf {x}})-\mathcal {S}({\textbf {x}}) +\mathcal {T}({\textbf {x}})\Vert _2^{2}. \end{aligned}$$

Here $\Vert \cdot \Vert _2$ denotes the $L^2$ norm. It is obvious that the problem (2) is a primal dual problem and then the alternating direction method can be used to solve it as follows.

$$\begin{aligned} \left\{ \begin{array}{lc} \mathcal {S}^{k+1}({\textbf {x}}) :=\displaystyle \mathop {\textrm{argmax}}\limits _{\mathcal {S}({\textbf {x}})\le C_{s}({\textbf {x}})} \mathcal {L}\left( \mathcal {S}({\textbf {x}}),\mathcal {T}^k({\textbf {x}}), {\textbf {q}}^k({\textbf {x}});w^k({\textbf {x}})\right) , & \qquad \qquad (3) \\ \mathcal {T}^{k+1}({\textbf {x}}) :=\displaystyle \mathop {\textrm{argmax}}\limits _{\mathcal {T}({\textbf {x}})\le C_{t}({\textbf {x}})} \mathcal {L}\left( \mathcal {S}^{k+1}({\textbf {x}}),\mathcal {T}({\textbf {x}}),{\textbf {q}}^k ({\textbf {x}});w^k({\textbf {x}})\right) , & \qquad \qquad (4)\\ {\textbf {q}}^{k+1}({\textbf {x}}) :=\displaystyle \mathop {\textrm{argmax}}\limits _{\Vert {\textbf {q}}({\textbf {x}})\Vert _{\infty }\le C_{q}({\textbf {x}})} \mathcal {L}\left( \mathcal {S}^{k+1}({\textbf {x}}),\mathcal {T}^{k+1}({\textbf {x}}),{\textbf {q}} ({\textbf {x}});w^k({\textbf {x}})\right) , & \qquad \qquad (5) \\ w^{k+1}({\textbf {x}})=w^k({\textbf {x}})-\lambda _1 \left( \textrm{div} {\textbf {q}}^{k+1}({\textbf {x}})-\mathcal {S}^{k+1}({\textbf {x}}) +\mathcal {T}^{k+1}({\textbf {x}})\right) , & \qquad \qquad (6) \\ \end{array} \right. \end{aligned}$$

where $\lambda _1>0$ is the penalty parameter and $C_{q}({\textbf {x}}):=C({\textbf {x}})\boldsymbol{\alpha }({\textbf {x}})$. We provide details for solving these sub-minimization problems as follows.

- To the subproblem (3), it is the constrain and convex optimization problem, we can use the gradient projection method to solve it by
  7 $$\begin{aligned} \mathcal {S}^{k+1}=\min \left\{ \mathcal {T}^k({\textbf {x}})+\textrm{div} {\textbf {q}}^{k} ({\textbf {x}})-\frac{w^{k}({\textbf {x}})}{\lambda _{1}}+\frac{1}{\lambda _{1}},C_{s}({\textbf {x}})\right\} . \end{aligned}$$
  Here $$C_{s}({\textbf {x}})=|I({\textbf {x}})-c_1*255 |$$.
- To the subproblems (4)-(5), they are the constrain and convex optimization problem. By using the gradient projection method, the solutions can be obtained from
  8 $$\begin{aligned} {\left\{ \begin{array}{ll} \mathcal {T}^{k+1}=\min \left\{ \mathcal {S}^{k+1}({\textbf {x}})-\textrm{div} {\textbf {q}}^{k}({\textbf {x}})+\frac{w^{k}({\textbf {x}})}{\lambda _{1}},C_{t}({\textbf {x}})\right\} ,\\ \textbf{q}^{k+1} =\min \Big \{\mathcal {A}^{k+1}({\textbf {x}}),C_{q}({\textbf {x}})\Big \}. \end{array}\right. } \end{aligned}$$

where ${C_{t}({\textbf {x}})=|I({\textbf {x}})-c_2*255 |}$, $\mathcal {A}^{k+1}({\textbf {x}})=\textbf{q}^{k}({\textbf {x}})+\sigma \nabla (\textrm{div} {\textbf {q}}^{k}({\textbf {x}})+\mathcal {T}^{k+1}({\textbf {x}})-\mathcal {S}^{k+1}({\textbf {x}}) -\frac{w^{k}({\textbf {x}})}{\lambda _{1}})$ and $\sigma>0$ is the step size.

More specifically, the numerical method of solving the model (1) can be summarized as follows.

**Algorithm 1:**
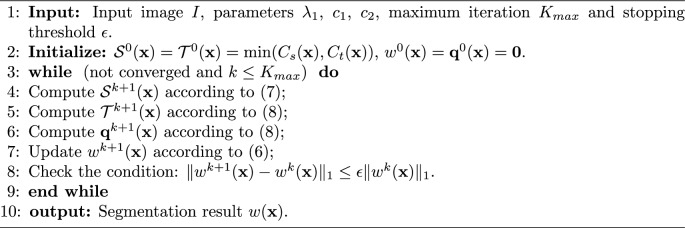
The algorithm to solve the weighted max-flow model (1).

#### Remark 2.1

There are several parameters in Algorithm 1 to be tuned in the numerical implementation. Here we choose them by trial-and-error method as maximum iteration $K_{max}=120$, iteration step $\sigma =0.25$, parameter $\lambda _1=0.25$, stopping threshold $\epsilon =5e-2$. In most cases, we set $c_{1}=0.35$, $c_{2}=1.00$, but it can be adjusted according to different data.

As demonstrated in the maximum flow scheme (1), the contour of the knee joint is typically delineated, encompassing the femur, the tibia, the patella, and the fibula. Nevertheless, to facilitate the visualization of the target area and assist the doctor in observing the lesion, the proposed segmentation scheme must extend from the bone in the cross-sectional, sagittal, and coronal planes. In instances where the image edge is indistinct, methodologies such as the closing and opening operations, which employ morphological operations, become essential. These operations involve dilation and erosion, respectively, of the image to address any deficiencies in the segmentation image.

### Watershed method

Adhesion between different joints is frequently observed in CT images of knee joints, particularly in cases of knee joint diseases. It is essential to note that certain segmentation regions may be linked after applying multiple morphology operators. In the developed scheme under consideration, the Distance Transform Watershed (DTW) proposed in^28^ is utilised. The fundamental principle of the watershed method is to conceptualise the image as a topological topography in geodesy. The grey value of each pixel in the image represents the altitude. Larger grey values characterise the regions exhibiting heightened brightness, while smaller grey values characterise the darker areas. The image contains one or more local minima in its grey value. For bone adhesion cases, we computed the Euclidean distance transform of the binary segmentation mask. The local maxima of this distance map serve as markers for the Watershed algorithm, which floods basins from these markers to separate connected regions (see Fig. 4). This approach is designed to prevent over-segmentation by constraining flooding to anatomically plausible boundaries.

### Manual interaction

Whilst the watershed method or the max-flow model ((1)) generally yields an accurate segmentation result, it is essential to note that certain drawbacks may still exist, such as over-segmentation, under-segmentation, and incorrect segmentation for edges with lower contrast. In such cases, the manual interaction scheme is utilised. Specifically, the term ’manual interaction’ refers to the process of manually enhancing the quality of segmentation through the adjustment of the method’s input parameters and subsequent post-processing of the results.

In the following section, an approach is outlined for the semi-automatic segmentation of knee CT images. This method is both accurate and effective, and is performed manually via a user interface. The manual interaction method is employed to introduce labelled seeds into the background, thereby demarcating the foreground from the background. In more detail, annotations are made on the area where two bones are connected close to each other, and the generated seed points are added to the background area as the initial constraint. Subsequently, the segmentation process is repeated, utilising the aforementioned weighted max-flow method. The variables employed in the subsequent model have equivalent significance to those in the model referenced as (1).

We label some image pixels as foreground seeds (i.e., $\Omega _{f}$) and background seeds (i.e., $\Omega _{b}$) as given prior supervision information. The indicator functions can be represented by

$$\begin{aligned} \delta _{\textrm{f}}(\textbf{x})= {\left\{ \begin{array}{ll} 1,\,\textbf{x}\in {\Omega }_{f}\\ 0,\,\textbf{x}\notin {\Omega }_{f} \end{array}\right. } \text{ and } \delta _{\textrm{b}}(\textbf{x})= {\left\{ \begin{array}{ll} 0,\,\textbf{x}\in \Omega _{b}\\ 1,\,\textbf{x}\notin \Omega _{b}. \end{array}\right. } \end{aligned}$$

Similarly, due to $\Omega _{f}$ and $\Omega _{b}$ are disjoint, we have

$$\begin{aligned} \delta _{\textrm{f}}(\Omega _{b})=0 \quad \text{ and }\quad \delta _{b}(\Omega _{f})=1. \end{aligned}$$

Here, we integrate max-flow-based segmentation with the given prior supervised constraint information. Then we extend the model (1) as

9 $$\begin{aligned} \begin{aligned} \displaystyle \max _{\mathcal {S}({\textbf {x}}),\mathcal {T}({\textbf {x}}), {\textbf {q}}({\textbf {x}})}\hspace{10pt}&\displaystyle \int _{\Omega }\delta _{\textrm{b}}(\textbf{x})\mathcal {S}({\textbf {x}})-\delta _{\textrm{f}}(\textbf{x}) \mathcal {T}({\textbf {x}})\textrm{d} {\textbf {x}},\\ \mathrm {s.t.}\hspace{30pt}&\mathcal {S}({\textbf {x}})\le C_{s}({\textbf {x}}),\hspace{3pt}\mathcal {T}({\textbf {x}})\le C_{t}({\textbf {x}}),\\&\Vert {\textbf {q}}({\textbf {x}})\Vert _{\infty }\le C({\textbf {x}})\boldsymbol{\alpha }({\textbf {x}}),\\&\textrm{div} {\textbf {q}}({\textbf {x}})-\mathcal {S}({\textbf {x}})+\mathcal {T}({\textbf {x}})=0. \end{aligned} \end{aligned}$$

To the equation constrain in the model (9), based on the Lagrangian multiplier method, we have the following saddle point problem

$$\begin{aligned} \begin{aligned} \displaystyle \max _{\mathcal {S}({\textbf {x}}),\mathcal {T}({\textbf {x}}),{\textbf {q}}({\textbf {x}})} \min _{v({\textbf {x}})}&\mathcal {L}(\mathcal {S}({\textbf {x}}),\mathcal {T}({\textbf {x}}), {\textbf {q}}({\textbf {x}});v({\textbf {x}})),\\ \mathrm {s.t.}&\mathcal {S}({\textbf {x}})\le C_{s}({\textbf {x}}),\mathcal {T}({\textbf {x}})\le C_{t}({\textbf {x}}),\\&\Vert {\textbf {q}}({\textbf {x}})\Vert _{\infty }\le C({\textbf {x}})\boldsymbol{\alpha }({\textbf {x}}). \end{aligned} \end{aligned}$$

where $v(\textbf{x})$ is the Lagrangian multiplier, and the augmented lagrangian function can be expressed as

$$\begin{aligned} \begin{aligned} \mathcal {L}_v(\mathcal {S}({\textbf {x}}),\mathcal {T}({\textbf {x}}), {\textbf {q}}({\textbf {x}});v({\textbf {x}})):&=\displaystyle \int _{\Omega }(\delta _{\textrm{b}}(\textbf{x}) -v({\textbf {x}}))\mathcal {S}({\textbf {x}}) +(v({\textbf {x}})-\delta _{\textrm{f}}(\textbf{x}))\mathcal {T}({\textbf {x}}) +v({\textbf {x}})\textrm{div} {\textbf {q}}({\textbf {x}})\textrm{d} {\textbf {x}}\\&-\frac{\lambda _{2}}{2}\Vert \textrm{div} {\textbf {q}}({\textbf {x}})-\mathcal {S}({\textbf {x}}) +\mathcal {T}({\textbf {x}})\Vert _2^{2}. \end{aligned} \end{aligned}$$

Under the framework of the alternating direction method of multipliers (ADMM), we have the iteration scheme as follows.

$$\begin{aligned} \left\{ \begin{array}{lc} \mathcal {S}^{k+1}({\textbf {x}}):=\displaystyle \mathop {\textrm{argmax}}\limits _{\mathcal {S}({\textbf {x}})\le C_{s}({\textbf {x}})} \mathcal {L}_v\left( \mathcal {S}({\textbf {x}}),\mathcal {T}^k({\textbf {x}}), {\textbf {q}}^k({\textbf {x}});v^k({\textbf {x}})\right) , & \qquad \qquad (10) \\ \mathcal {T}^{k+1}({\textbf {x}}):=\displaystyle \mathop {\textrm{argmax}}\limits _{\mathcal {T}({\textbf {x}})\le C_{t}({\textbf {x}})} \mathcal {L}_v\left( \mathcal {S}^{k+1}({\textbf {x}}),\mathcal {T}({\textbf {x}}),{\textbf {q}}^k ({\textbf {x}});v^k({\textbf {x}})\right) , & \qquad \qquad (11) \\ {\textbf {q}}^{k+1}({\textbf {x}}):=\displaystyle \mathop {\textrm{argmax}}\limits _{\Vert {\textbf {q}}({\textbf {x}})\Vert _{\infty }\le C_{q}({\textbf {x}})} \mathcal {L}_v\left( \mathcal {S}^{k+1}({\textbf {x}}),\mathcal {T}^{k+1}({\textbf {x}}),{\textbf {q}} ({\textbf {x}});v^k({\textbf {x}})\right) , & \qquad \qquad (12) \\ v^{k+1}({\textbf {x}})=v^k({\textbf {x}})-\lambda _2 \left( \textrm{div} {\textbf {q}}^{k+1}({\textbf {x}})-\mathcal {S}^{k+1}({\textbf {x}}) +\mathcal {T}^{k+1}({\textbf {x}})\right) ,& \qquad \qquad (13) \\ \end{array} \right. \end{aligned}$$

where $\lambda _2>0$ is the penalty parameter, and $C_{q}({\textbf {x}}):=C({\textbf {x}})\boldsymbol{\alpha }({\textbf {x}})$. To the subproblems (10)-(13), we can use the similar method as solving (3)-(5) to solve them. Specifically, their solution can be obtained by

$$\begin{aligned} \left\{ \begin{array}{lc} \mathcal {S}^{k+1}({\textbf {x}})=\min \left\{ \mathcal {T}^k({\textbf {x}})+\textrm{div} {\textbf {q}}^{k}({\textbf {x}})-\frac{v^{k}({\textbf {x}})}{\lambda _{2}}+\frac{\delta _{\textrm{b}}({\textbf {x}})}{\lambda _{2}},C_{s}({\textbf {x}})\right\} . & \qquad \qquad (14) \\ \mathcal {T}^{k+1}({\textbf {x}})=\min \left\{ \mathcal {S}^{k+1}({\textbf {x}})-\textrm{div} {\textbf {q}}^{k}({\textbf {x}})+\frac{v^{k}({\textbf {x}})}{\lambda _{2}}-\frac{\delta _{\textrm{f}}({\textbf {x}})}{\lambda _{2}},C_{t}({\textbf {x}})\right\} & \qquad \qquad (15) \\ {\textbf {q}}^{k+1}({\textbf {x}})=\min \Big \{\mathcal {A}_v^{k+1}({\textbf {x}}),C_{q}({\textbf {x}})\Big \}. & \qquad \qquad (16) \\ \end{array} \right. \end{aligned}$$

where $C_{s}({\textbf {x}})=|I({\textbf {x}})-c_1*255 |$, ${ C_{t}({\textbf {x}})=|I({\textbf {x}})-c_2*255 |}$, $\mathcal {A}_v^{k+1}({\textbf {x}})=\textbf{q}^{k}({\textbf {x}})+\sigma \nabla (\textrm{div} {\textbf {q}}^{k}({\textbf {x}})+\mathcal {T}^{k+1}({\textbf {x}})-\mathcal {S}^{k+1}({\textbf {x}}) -\frac{v^{k}({\textbf {x}})}{\lambda _{1}})$ and $\sigma>0$ is the step size. Based on (14)-(16), we have following algorithm 2 to solve the problem (9). For the parameter settings of this part, we choose the same setting as did in Remark 2.1.

**Algorithm 2:**
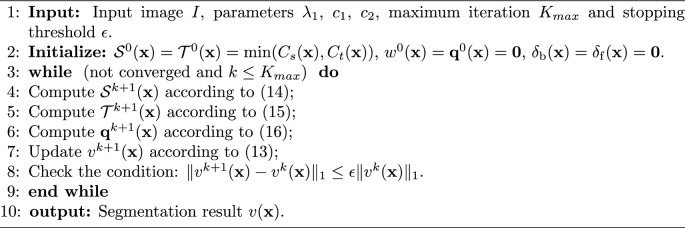
The algorithm to solve the weighted max-flow model (9).

### 3D discretization

All input CT volumes are resampled to isotropic voxels in order to ensure consistent spatial relationships and distance measures. In the experiments reported here, a working resolution of 1.0 $\times$ 1.0 $\times$ 1.0 mm is employed. Following the resampling process, each voxel is treated as a node in a graph, with the segmentation variables defined on the resulting voxel grid. It is essential to note that boundary voxels are connected exclusively to in-domain neighbors, thereby ensuring the accurate definition of boundary behavior. To minimize superfluous computation and memory utilization, the execution of processes is confined to a compact voxel subdomain centered on an initial region of interest (ROI) when relevant. The implementation choices outlined hereinafter serve to eliminate directional bias in distance transforms, thereby ensuring the subsequent clarity and ambiguity of 3D graph operations and marker propagation.

### Neighborhood definition

The graph topology employs a six-connected cubic neighbourhood (also known as a 6-connected neighbourhood or face adjacency) as the default configuration. In the isotropic voxel grid, this corresponds to a unit Euclidean distance between connected voxel centres. The pair edges are thus constructed according to this fixed topology, thereby producing a sparse graph that achieves a balance between computational efficiency and segmentation fidelity for bone structures in CT.

### Seeding strategy and unary construction

The seeding strategy utilises sparse three-dimensional annotations, wherein a typical number of 2–6 key slices per case are annotated with foreground/background marks. These marks are then propagated in 3D. Seeds may be enforced as hard constraints by assigning infinite (or sufficiently large) unary costs to fix a voxel label, or treated as soft priors by assigning high but finite unary penalties that express probabilistic confidence. In order to convert sparse point, line, and plane annotations into a dense unary field, it is necessary to expand each seed using a distance-based decay (e.g., a Gaussian radial decay or fixed-radius assignment). This process ensures that nearby voxels receive strong prior weights, while those at greater distances receive progressively weaker priors. The resulting unary field is then combined with image-based likelihoods and utilised in the energy above.

### Workflow

**Fig. 2:**
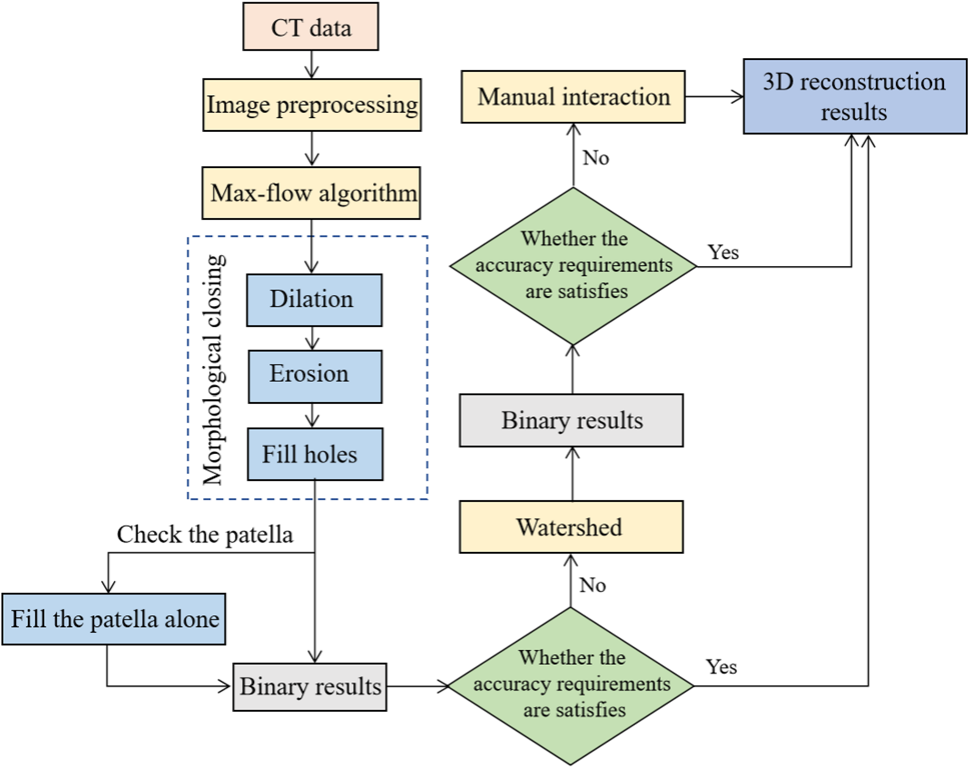
The workflow of the knee joint segmentation scheme.

We present the workflow that allows the semi-automated segmentation of knee CT images, as shown in Fig. 2. In the following discussion, we aim to present and analyse two logical judgments. If optimal segmentation is achieved directly, 3D reconstruction can proceed. Otherwise, the watershed algorithm and manual interaction are employed as post-processing steps to refine the results.

## Results

The 18 datasets comprise a range of pathologies (e.g., osteoarthritis, fractures) and scanning protocols (slice thickness: 0.6–2mm), which collectively encompass typical clinical scenarios. The statistical significance of these results was confirmed by the low standard deviations observed in the metrics presented in Table 2. This section outlines the specific technique used in our segmentation scheme for knee CT images. The experimental data have been sourced from the following website: https://github.com/mkrcah/bone-segmentation. A seasoned professional from Luoyang Orthopedic Hospital of Henan Province(Orthopedic Hospital of Henan Province) has labeled the ground truth of the image data. Table 1 displays the fundamental data and a series of knee CT images. All experiments are performed using the software MATLAB (R2021a) on a Windows (10) (64-bit) desktop computer with an Intel Core i7 3.20 GHz processor and 16.0 GB of RAM.

**Table 1:** Two groups of data in the knee CT datasets.

First	# 1	(512*512*150)	# 9	(512*512*232)
	# 2	(512*512*166)	# 10	(512*512*214)
	# 3	(512*512*172)	# 11	(512*512*218)
	# 4	(512*512*221)	# 12	(512*512*217)
	# 5	(512*512*123)	# 13	(512*512*214)
	# 6	(512*512*230)	# 14	(215*124*452)
	# 7	(512*512*163)	# 15	(210*169*478)
	# 8	(512*512*241)	# 16	(179*147*503)
Second	# 17	(512*512*187)	# 18	(512*512*84)

**Table 2:** Performance metrics for medical segmentation software and our knee joint segmentation system.

Images	MITK$\vphantom{0}_{1}$	MITK$\vphantom{0}_{2}$	3D Slicer	ImageJ	Seg3D	Ours	MITK$\vphantom{0}_{1}$	MITK$\vphantom{0}_{2}$	3D Slicer	ImageJ	Seg3D	Ours
# 1	0.7991	0.7355	0.9161	0.7686	0.8346	0.9436	0.8883	0.8476	0.9563	0.8692	0.9099	0.9710
# 2	0.6978	0.6598	0.9265	0.6747	0.7181	0.9354	0.8220	0.7950	0.9618	0.8057	0.8359	0.9666
# 3	0.9229	0.8398	0.9420	0.7603	0.9226	0.9451	0.9599	0.9129	0.9701	0.8638	0.9597	0.9718
# 4	0.5193	0.5105	0.9096	0.7529	0.5186	0.9094	0.6835	0.6760	0.9526	0.8590	0.6830	0.9526
# 5	0.6656	0.7388	0.9048	0.7090	0.7324	0.9229	0.7993	0.8498	0.9500	0.8298	0.8455	0.9599
# 6	0.7828	0.5326	0.9385	0.6246	0.7828	0.9370	0.8782	0.6950	0.9683	0.7689	0.8781	0.9675
# 7	0.6698	0.6859	0.9231	0.5875	0.7090	0.9289	0.8022	0.8137	0.9600	0.7402	0.8297	0.9632
# 8	0.6591	0.4669	0.9354	0.3851	0.6606	0.9394	0.7945	0.6365	0.9666	0.5561	0.7956	0.9688
# 9	0.6745	0.4335	0.8862	0.8070	0.6967	0.9373	0.8056	0.6048	0.9397	0.8932	0.8212	0.9676
# 10	0.8532	0.5669	0.8961	0.8642	0.8605	0.9317	0.9208	0.7317	0.9452	0.9272	0.9250	0.9646
# 11	0.8439	0.6002	0.9463	0.8195	0.8217	0.9468	0.9154	0.7502	0.9724	0.9008	0.9021	0.9727
# 12	0.5998	0.3203	0.9170	0.8535	0.6430	0.9260	0.7498	0.4852	0.9567	0.7444	0.7827	0.9616
# 13	0.8593	0.5893	0.9525	0.9017	0.8951	0.9143	0.9242	0.7416	0.9757	0.9483	0.9447	0.9553
# 14	0.6353	0.4545	0.8575	0.5471	0.8646	0.9085	0.7770	0.6249	0.9233	0.7073	0.9274	0.9521
# 15	0.9453	0.4781	0.9169	0.8343	0.9472	0.9541	0.9719	0.6469	0.9566	0.9097	0.9729	0.9765
# 16	0.8833	0.3343	0.7879	0.6900	0.8967	0.9033	0.9308	0.5010	0.8814	0.8166	0.9455	0.9492
Mean	0.7507	0.5592	0.9098	0.7238	0.7815	**0.9302**	0.8515	0.7071	0.9523	0.8213	0.8724	**0.9638**
Std	0.1224	0.1422	0.0392	0.1320	0.1154	**0.0146**	0.0807	0.1183	0.0224	0.0972	0.0761	**0.0079**
	Precision						Recall					
# 1	0.8425	0.7712	0.9469	0.9914	0.8914	0.9570	0.9395	0.9408	0.9657	0.7738	0.9291	0.9854
# 2	0.8173	0.9895	0.9815	0.9354	0.9808	0.9871	0.8268	0.6644	0.9397	0.7639	0.7283	0.9470
# 3	0.9467	0.9679	0.9770	0.9939	0.9653	0.9862	0.9735	0.8639	0.9634	0.8000	0.9542	0.9577
# 4	0.9613	0.9648	0.9632	0.9274	0.9615	0.9732	0.5302	0.5202	0.9423	0.7421	0.5296	0.9328
# 5	0.8144	0.9454	0.9529	0.9409	0.9333	0.9764	0.7847	0.7717	0.9471	0.8155	0.7729	0.9439
# 6	0.9312	0.5402	0.9616	0.7274	0.9469	0.9838	0.8308	0.9743	0.9750	0.7876	0.8187	0.9517
# 7	0.8571	0.9770	0.9510	0.6981	0.9653	0.9794	0.7540	0.6972	0.9692	0.3938	0.7275	0.9475
# 8	0.9636	0.4726	0.9567	0.9457	0.9582	0.9832	0.6759	0.9746	0.9767	0.8078	0.6802	0.9548
# 9	0.8892	0.4392	0.9658	0.9989	0.9495	0.9771	0.7364	0.9706	0.9149	0.8832	0.7235	0.9583
# 10	0.9275	0.5922	0.9633	0.9757	0.9658	0.9827	0.9142	0.9571	0.9278	0.8766	0.8875	0.9293
# 11	0.9708	0.6088	0.9713	0.9264	0.9779	0.9867	0.8659	0.9771	0.9736	0.7444	0.8373	0.9590
# 12	0.7786	0.3218	0.9555	0.9999	0.9774	0.9828	0.7231	0.9854	0.9579	0.9229	0.6527	0.9413
# 13	0.8978	0.5929	0.9899	0.9751	0.9700	0.9449	0.9523	0.9898	0.9619	0.6341	0.9206	0.9852
# 14	0.6397	0.4557	0.8908	0.7996	0.9138	0.9316	0.9891	0.9941	0.9582	0.8409	0.9413	0.9735
# 15	0.9804	0.4783	0.9409	0.9907	0.9806	0.9587	0.9635	0.9990	0.9729	0.7473	0.9653	0.9950
# 16	0.9254	0.3344	0.8072	0.9901	0.9354	0.9578	0.9510	0.9983	0.9705	0.7738	0.9559	0.9408
Mean	0.8840	0.6532	0.9485	0.9260	0.9546	**0.9718**	0.8382	0.8924	**0.9573**	0.7692	0.8140	0.9565
Std	0.0872	0.2365	0.0422	0.0938	0.0246	**0.0162**	0.1261	0.1433	**0.0176**	0.1171	0.1272	0.0187
	Specificity						Accuracy					
# 1	0.9909	0.9869	0.9969	0.9995	0.9937	0.9974	0.9884	0.9848	0.9953	0.9836	0.9903	0.9968
# 2	0.9978	0.9999	0.9998	0.9992	0.9998	0.9998	0.9958	0.9939	0.9991	0.9946	0.9954	0.9992
# 3	0.9973	0.9986	0.9989	0.9997	0.9983	0.9993	0.9962	0.9922	0.9972	0.9853	0.9962	0.9973
# 4	0.9991	0.9991	0.9991	0.9983	0.9991	0.9994	0.9793	0.9785	0.9978	0.9929	0.9792	0.9977
# 5	0.9935	0.9981	0.9984	0.9979	0.9977	0.9992	0.9863	0.9888	0.9966	0.9870	0.9886	0.9973
# 6	0.9971	0.9809	0.9984	0.9885	0.9977	0.9993	0.9895	0.9808	0.9974	0.9823	0.9893	0.9973
# 7	0.9962	0.9994	0.9987	0.9920	0.9991	0.9995	0.9890	0.9884	0.9979	0.9873	0.9897	0.9981
# 8	0.9992	0.9886	0.9990	0.9988	0.9991	0.9996	0.9893	0.9884	0.9986	0.9677	0.9895	0.9986
# 9	0.9995	0.9756	0.9986	0.9999	0.9977	0.9990	0.9817	0.9755	0.9950	0.9898	0.9824	0.9972
# 10	0.9973	0.9849	0.9987	0.9991	0.9987	0.9979	0.9942	0.9843	0.9961	0.9944	0.9943	0.9974
# 11	0.9986	0.9812	0.9986	0.9964	0.9989	0.9993	0.9916	0.9811	0.9974	0.9905	0.9901	0.9974
# 12	0.9951	0.9853	0.9990	0.9999	0.9995	0.9996	0.9888	0.9853	0.9981	0.9926	0.9883	0.9983
# 13	0.9960	0.9844	0.9996	0.9990	0.9988	0.9993	0.9945	0.9845	0.9982	0.9960	0.9957	0.9967
# 14	0.9622	0.9439	0.9882	0.9775	0.9906	0.9922	0.9639	0.9462	0.9854	0.9349	0.9859	0.9904
# 15	0.9977	0.9428	0.9931	0.9989	0.9977	0.9950	0.9941	0.9456	0.9911	0.9795	0.9943	0.9950
# 16	0.9913	0.9282	0.9780	0.9881	0.9925	0.9952	0.9873	0.9306	0.9774	0.9579	0.9888	0.9895
Mean	0.9943	0.9799	0.9964	0.9958	0.9974	**0.9982**	0.9881	0.9768	0.9949	0.9823	0.9899	**0.9965**
Std	0.0087	0.0215	0.0056	0.0061	0.0026	**0.0021**	0.0078	0.0182	0.0056	0.0157	0.0046	**0.0026**

### Compare experimental results and analysis

In order to facilitate a fair comparison, all methods (including the present one) were tested in fully automatic mode, without the use of manual seeds. The manual interaction module was exclusively activated in cases classified as severe (see Table 3), which were excluded from comparisons presented in Table 2. To demonstrate the reasonableness of the proposed scheme, numerical comparisons are presented for 18 groups of 3D knee CT images. The main parameters of the system are $c_1$ and $c_2$, as well as the range of HU. As demonstrated in the preceding experimental data, the parameter settings employed were $c_1 = 0.35$ and $c_2 = 1.00$. The range of HU was established as [300, 1600]. These components are designed to be fixed and do not require manual adjustment. These datasets do not require supervision information, i.e., interactive processing, interactive processing is not required. Four different kinds of open-source medical software, including MITK^29^, 3D Slicer^30^, ImageJ^31^, and Seg3D (www.sci.utah.edu/cibc/software), are to be used. The following section details the software utilised in the numerical comparisons.

- ***MITK***: The Medical Imaging Interaction Toolkit primarily offers four segmentation methods, including Upper and Lower (UL) Threshold, Otsu, Region Growth segmentation, and Fast Marching Method. This paper primarily employs the UL Threshold and Otsu methods for use in the numerical comparisons. More specifically, we use the UL Threshold method (designated as $\textrm{MITK}_{1}$) and Otsu (designated as $\textrm{MITK}_{2}$) to segment the data, and then fill in the results of the segmentation accordingly.
- ***3D Slicer***: Here we mainly use a semi-automatic segmentation method of the 3D Slicer. To be specific, non-target areas can be removed by adjusting the upper and lower parameters of the threshold using the scissors tool in the 3D Slicer toolbar. After obtaining the original segmentation image, we use the morphological method to fill the hole in the inner region.
- ***ImageJ***: It includes many semi-automatic segmentation methods. Here, we employ the Weka segmentation to deal with the knee CT images. For Trainable Weka Segmentation 3D^32^, a set of pixel samples is defined and represented as feature vectors, and a Weka learning scheme is trained on these samples, ultimately applied to classify the remaining image data. However, using the Weka environment to optimize parameters for large datasets is time-consuming.
- ***Seg3D***: It mainly includes some threshold methods. The main steps of this scheme are, firstly, obtaining the segment results and then using the morphological method to fill the holes in the segmentation region.

To facilitate a comparative analysis of segmentation quality, several indices have been selected to quantify the effectiveness of the segmentation. The indices selected for this purpose include Precision, Recall^33^, Jaccard Similarity (JS)^34^, Dice Similarity Coefficient (DSC)^34^, Accuracy^35^, and Specificity^36^.

**Fig. 3:**
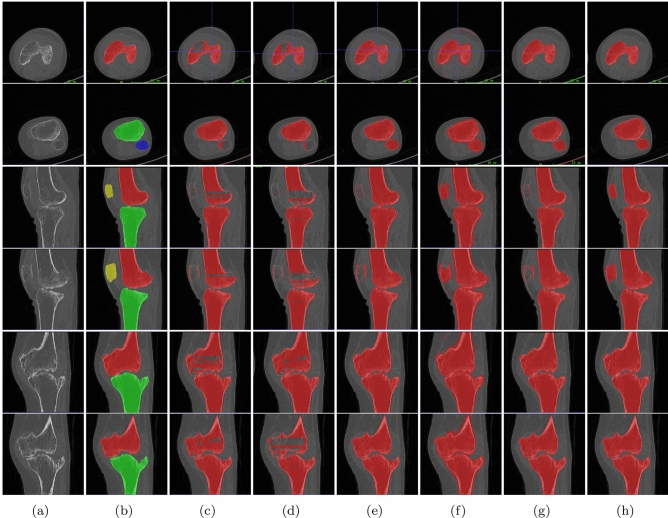
(**a**) Original CT data. (**b**) Ground truth, which show different colors due to different pixel markers. (**c**) UL Threshold segmentation method by the MITK. (**d**) Otsu segmentation method by the MITK. (**e**) The threshold segmentation method and scissors tool by the 3D Slicer. (**f**) Trainable Weka Segmentation 3D method by the ImageJ. (**g**) Seg3D. (**h**) Ours.

In the context of the initial dataset, the efficacy of the proposed scheme is demonstrated by its ability to achieve accurate segmentation of the femur and tibia without requiring any supervisory information. However, the reviewed medical software did not achieve efficient segmentation. As illustrated in Fig. 3, two slices have been selected at random to demonstrate the segmentation outcomes of diverse methodologies from three distinct viewpoints. The primary objective of this study is to determine the efficacy of the experimental results in accurately removing unrelated tissue and to assess the accuracy of bone edge segmentation. Comparative analysis with ground truth data reveals that the open-source software tested was more susceptible to over-segmentation and under-segmentation than the proposed method (Fig. 3). The proposed scheme has the capacity to integrate seamlessly with the existing framework. As demonstrated in Table 2, the proposed scheme successfully attains an optimal sensitivity index between the segmented and ground truth data on volume-based measures.

**Table 3:** Display of data sets metrics about systemic segmentation of severe lesions.

Images	Precision	Recall	JS	DSC	Specificity	Accuracy
# 17	0.9305	0.9737	0.9077	0.9516	0.9989	0.9985
# 18	0.9867	0.9556	0.9435	0.9709	0.9998	0.9993
Mean	0.9586	0.9647	0.9256	0.9613	0.9994	0.9989
Std	0.0281	0.0091	0.0179	0.0096	0.0005	0.0004

**Fig. 4:**
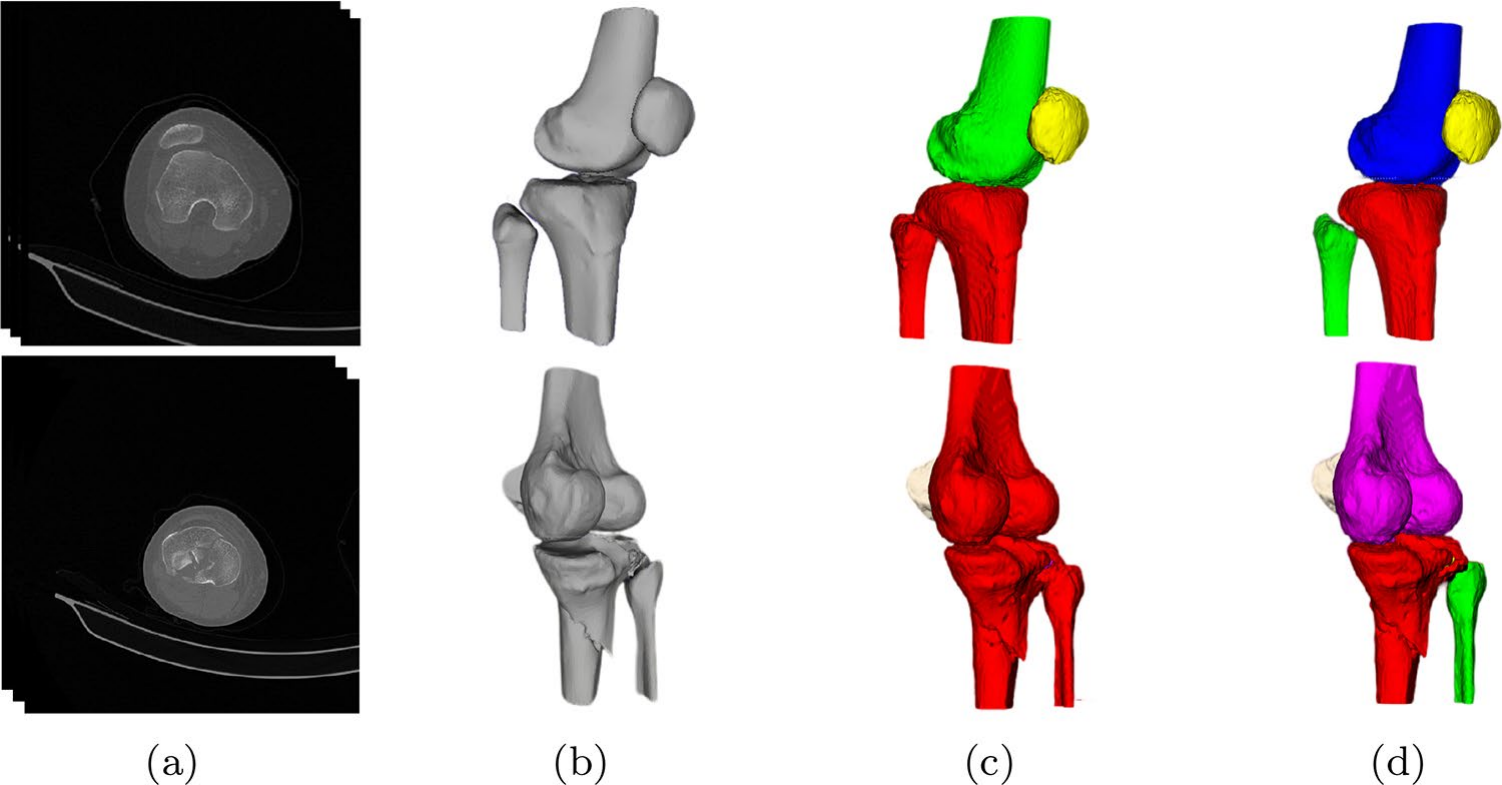
The original knee CT images, ground truth and segmentation results are displayed in 3D. (a) Original CT data. (b) Ground truth. (c) The segmentation results without supervision information. (d) The segmentation results with supervision information. The first row of data is tibia-fibula adhesion. The second row of data is the adhesion of femur, tibia and fibula.

The second group of images exhibits lower image quality and higher pathology. The ambiguity of the delineation poses a significant challenge to the accurate segmentation process. The fully automatic segmentation resulted in the merging of different knee joint tissues comprising the knee joint images, including the femur, tibia, fibula, and patella. The subsequent analysis will thus be based on the segmentation results obtained from the proposed scheme, facilitated by the utilisation of interactive methodologies. Furthermore, a comparison with the ground truth has been made, resulting in the arrangement of the segmentation indicators in Table 3 as demonstrated in Figure 4. Two illustrative examples are presented: one concerning the adhesion of tibia and fibula, and the other about the adhesion of femur, tibia, and fibula. Uniform colors indicate interconnected areas; otherwise, areas are displayed in different colors..

To evaluate the consistency of three-dimensional segmentation, a focused analysis was conducted on two cases that exhibited severe interslice adhesion and were of a clinical nature. As illustrated in Fig. 4a, Case 17 exhibited tibia-fibula fusion across 45 contiguous axial slices. Conversely, as demonstrated in Fig. 4b, Case 18 presented with femur-tibia adhesion spanning 32 slices. The maximum flow formulation successfully resolved complex anatomical adhesions in three dimensions. The initialisation process required only 2–3 strategically positioned seed points on key mid-sagittal slices, thereby demonstrating the efficient axial propagation of segmentation contours without observable staircase artefacts at 1 mm slice intervals (Fig. 4c-d).

## The graphical user interface

**Fig. 5:**
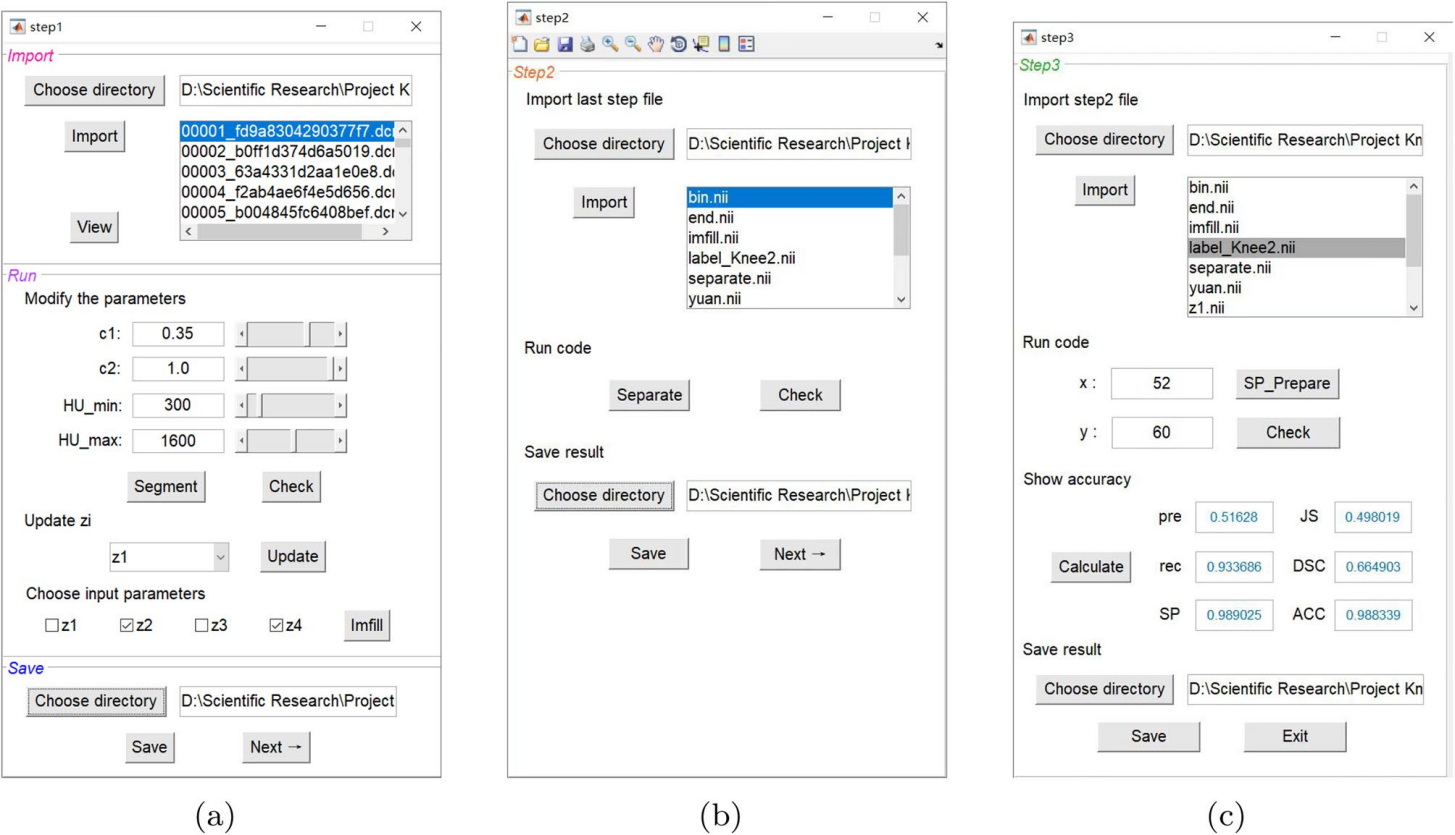
The GUI of our proposed scheme to segment knee joint CT images, (**a**) Step I: Bone Segmentation, (**b**) Step II: Post-Processing, (**c**) Step III: Manual Interaction.

As demonstrated in Fig. 5, the GUI for the proposed scheme has been designed using the aforementioned methodology. The system has a straightforward interface and simple operation, divided into three steps. The scheme comprises a comprehensive module that encompasses the reading of data to obtain the segmentation result. Additionally, it incorporates a range of interaction methods to facilitate the management of complex segmentation tasks. If the initial segmentation result from Step I is satisfactory, the 3D reconstruction can be generated directly. Otherwise, users proceed to Step II for post-processing. It is evident that the initial Step of the process, designated as Step I, is sufficient to fulfil the stipulated criteria for the majority of the data.

### Step I: Bone segmentation

Step I in this process can be subdivided into two constituent parts: data preprocessing and image segmentation. The objective of data preprocessing is to adjust the HU interval in order to enhance the robustness of the segmentation. As the data acquired from knee CT scans may contain irrelevant tissue, the Check button on the segmentation result is used to verify the bone tissues and select the bone tissues to be segmented, thereby removing irrelevant tissues. Furthermore, a bespoke treatment plan for the patella of the knee joint is available. The patella is understood to satisfy the morphological convexity. Accordingly, a separate algorithm has been designed to ensure that the patella is filled satisfactorily.

### Step II: Post-processing

The following data are indicative of cases involving severe adhesions of the femur and tibia, or adhesions between the tibia and fibula. The subsequent Step involves the implementation of the watershed algorithm, a process designed to address the challenges posed by significant adhesion in data sets.

### Step III: Manual interaction

In Step III, the manual interaction method is employed. The result data from Step II is then entered according to the button prompts. Subsequently, the result data from Step II is manually checked, and the knee CT slices that are not divided are filtered out. The number of layers requiring manual interaction is then entered into the scheme, and the run button is subsequently clicked. Step III incorporates a numerical evaluation module that displays the quantitative values of the segmentation results in comparison with the ground truth, utilizing various evaluation indices. Manual interaction is only required for a subset of slices (typically < 0.05 of total slices) where severe adhesion occurs. Users can annotate key slices, and the algorithm propagates corrections to adjacent slices via 3D max-flow.

## Discussions

Accurate segmentation of three-dimensional (3D) knee joint computed tomography (CT) images remains a critical technological bottleneck in advancing digital orthopaedic diagnostics towards intelligent, standardised clinical pathways. The task at hand is predicated on three fundamental principles: firstly, high algorithmic robustness is requisite; secondly, precise anatomical boundary delineation is necessary; and thirdly, seamless clinical integration is essential. However, contemporary mainstream segmentation approaches often demonstrate deficiencies when processing images with low signal-to-noise ratio (SNR), primarily due to their limited ability to discern weak inter-tissue boundaries and their excessive reliance on manual intervention. These constraints substantially compromise their viability for routine clinical deployment. To overcome these challenges, a novel segmentation framework is proposed that combines an adaptive weighted continuous max-flow model with a distance transform-guided watershed algorithm. This integrated approach is designed to achieve accurate segmentation of anatomically complex structures while maintaining a practical balance between automation and user-guided refinement. The proposed framework offers significant performance improvements enabled by three key innovations: 1. Gradient-Sensitive Energy Modelling: The present study introduces a continuous flow energy function enhanced with gradient-sensitive, adaptively weighted boundary terms. This formulation has been demonstrated to enhance responsiveness to subtle anatomical boundaries, such as those located at the tibiofibular junction, thereby enhancing tissue interface localization at the fundamental energy formulation level. 2. Modular Clinical-Workflow-Oriented GUI: A three-tier modular graphical user interface (GUI) has been developed, aligned with standard clinical segmentation workflows. The software encompasses preprocessing, automatic segmentation, post-processing, and optional interactive refinement modules. This design facilitates flexible user control over intervention levels and optimises segmentation efficiency across use cases.

To further optimize the trade-off between segmentation accuracy and processing efficiency, a hierarchical interaction strategy is implemented. Semi-supervised seed annotation is selectively applied to approximately $15\%$ of the more challenging cases, such as those with severe anatomical adhesion or ambiguous cartilage interfaces (see Table 2). At the same time, the remainder are processed via a fully automated pipeline. This stratified approach ensures the efficient handling of routine cases while enabling precision adjustments in pathological edge cases. Despite the potential for inter-operator variability arising from manual intervention, the implementation of standardised training protocols and quality assurance measures has effectively constrained such variations within clinically acceptable limits (DSC < 0.05).

Despite these substantial advancements, several avenues for further research remain: Firstly, the extension to multi-modality imaging is to be considered. The current framework has been optimised for CT imaging. Subsequent studies will investigate cross-modality generalization, with a particular focus on MRI, through cross-modality adaptation techniques. Secondly, the subject of automated parameter optimization is addressed. It is evident that several critical hyperparameters, including regularization weights, are currently being tuned empirically. The ongoing development of a meta-learning framework aims to enable data-driven parameter selection based on image texture features.

## Conclusions

The proposed methodology outlines a novel approach to accurately segmenting knee joint CT images, following a rigorous data preprocessing step. The scheme was divided into three modules to accommodate the diverse data, and it can also be operated or stopped artificially at the end of each module to achieve the desired effect. The present study employed six distinct evaluation indicators to ascertain the relative effectiveness of the proposed scheme in comparison to four alternative open-source software solutions. The numerical results obtained demonstrated that the proposed scheme was capable of achieving satisfactory outcomes. To verify the generalization ability of the proposed scheme, the segmentation of additional fracture data was also considered. It was found that the proposed scheme still achieved satisfactory results.

## Data Availability

The knee joint CT data used in this study (from 18 patients) have been securely preserved by the corresponding author (cxy1260@126.com). Researchers interested in accessing these data for academic or scientific purposes may contact the corresponding author via email. Data will be shared on reasonable request and evaluated on a case-by-case basis.
